# Laparoscopic Removal of an Ingested Foreign Body with Transesophageal Migration into the Mediastinum

**DOI:** 10.7759/cureus.2712

**Published:** 2018-05-30

**Authors:** Nagaraj Kapil, Raja Kalayarasan, Pottakkat Biju, Chandrasekar Sandip, Gnanasekaran Senthil

**Affiliations:** 1 Surgical Gastroenterology, Jawaharlal Institute of Postgraduate Medical Education and Research (JIPMER), Puducherry, IND

**Keywords:** mediastinum, foreign body, laparoscopy, transesophageal migration

## Abstract

Removal of a transesophageal migrated foreign body is recommended to prevent injury to adjacent structures. As the endoscopic approach is not feasible for a transesophageal foreign body migrated into the mediastinum, the thoracoscopic approach is recommended. The thoracoscopic approach often requires single lung ventilation and is associated with more pulmonary complications. The use of a laparoscopic approach to remove a mediastinal foreign body has not been reported earlier. In this report, the authors describe a laparoscopic approach for the removal of a transesophageal migrated foreign body into the lower mediastinum.

## Introduction

Foreign body ingestion is most common in children aged between six months and six years [1]. In adults, foreign body impactions are mostly accidental or occur in the context of a pre-existing pathology. Common foreign bodies ingested by adults are food items (fishbone / meat bone) or dentures. The clinical presentation is usually immediate with acute onset odynophagia and dysphagia. A few patients may also present with complications such as perforation and mediastinitis that are associated with high morbidity and mortality even after surgical intervention. Late presentations are infrequent, with transesophageal migration being one of them. Transesophageal migration of a foreign body is an indication for surgical removal as it is associated with devastating complications like aortoesophageal and aortopulmonary fistulas [2-6]. In the literature, it is seen that a transthoracic approach (thoracotomy or thoracoscopy) is commonly used to remove a foreign body in the mediastinum [7-9]. As the transthoracic approach often requires single lung ventilation, it is associated with more pulmonary complications. The laparoscopic approach is associated with lesser pulmonary complications compared to the transthoracic approach. To our knowledge, this is the first report of the laparoscopic approach being used in the removal of a transesophageal migrated foreign body.

## Case presentation

**Figure 1:**
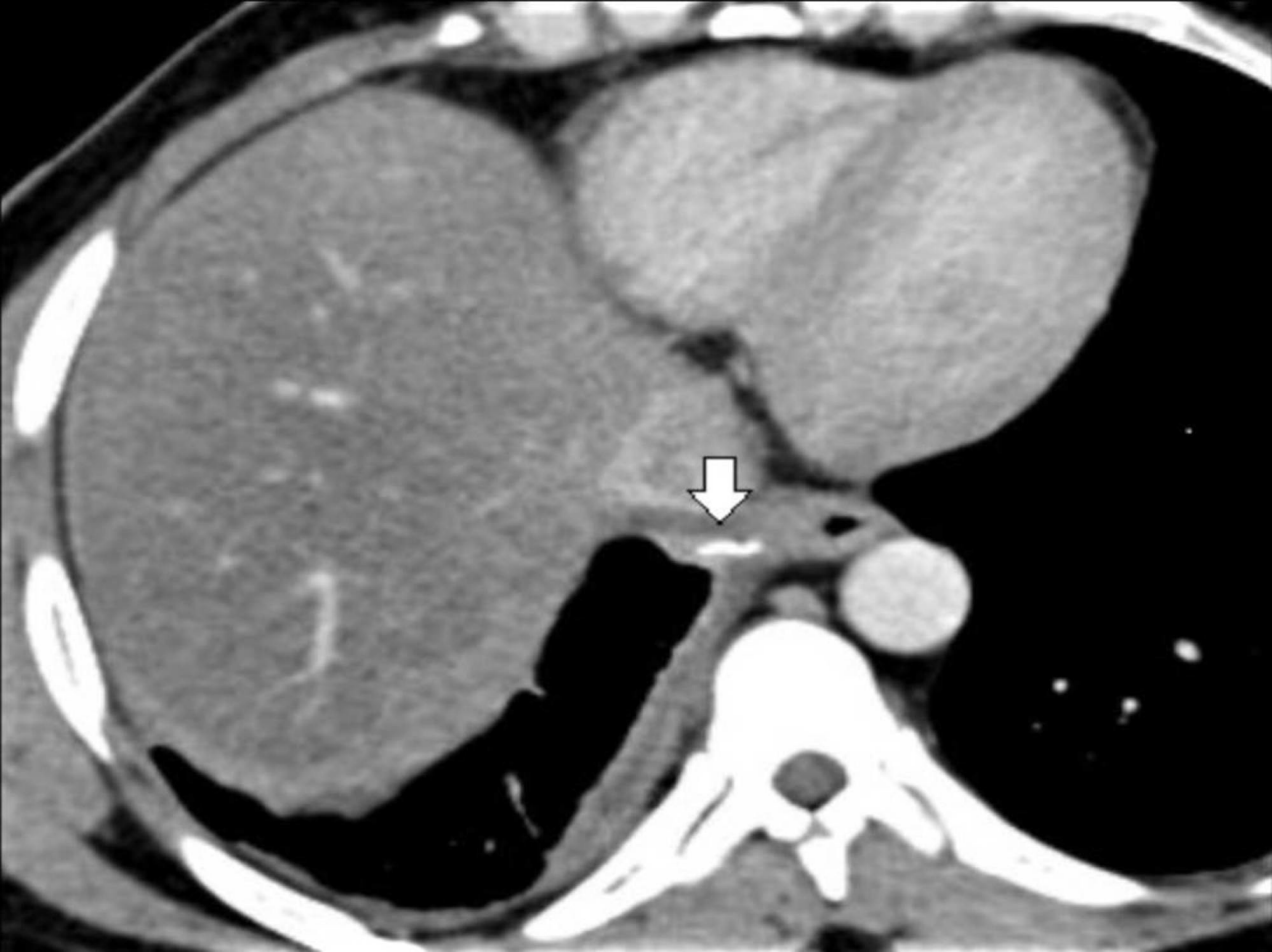
Axial section showing radio opaque foreign body in mediastinum. Note the proximity to inferior vena cava and the adjacent lung (white arrow)

**Figure 2:**
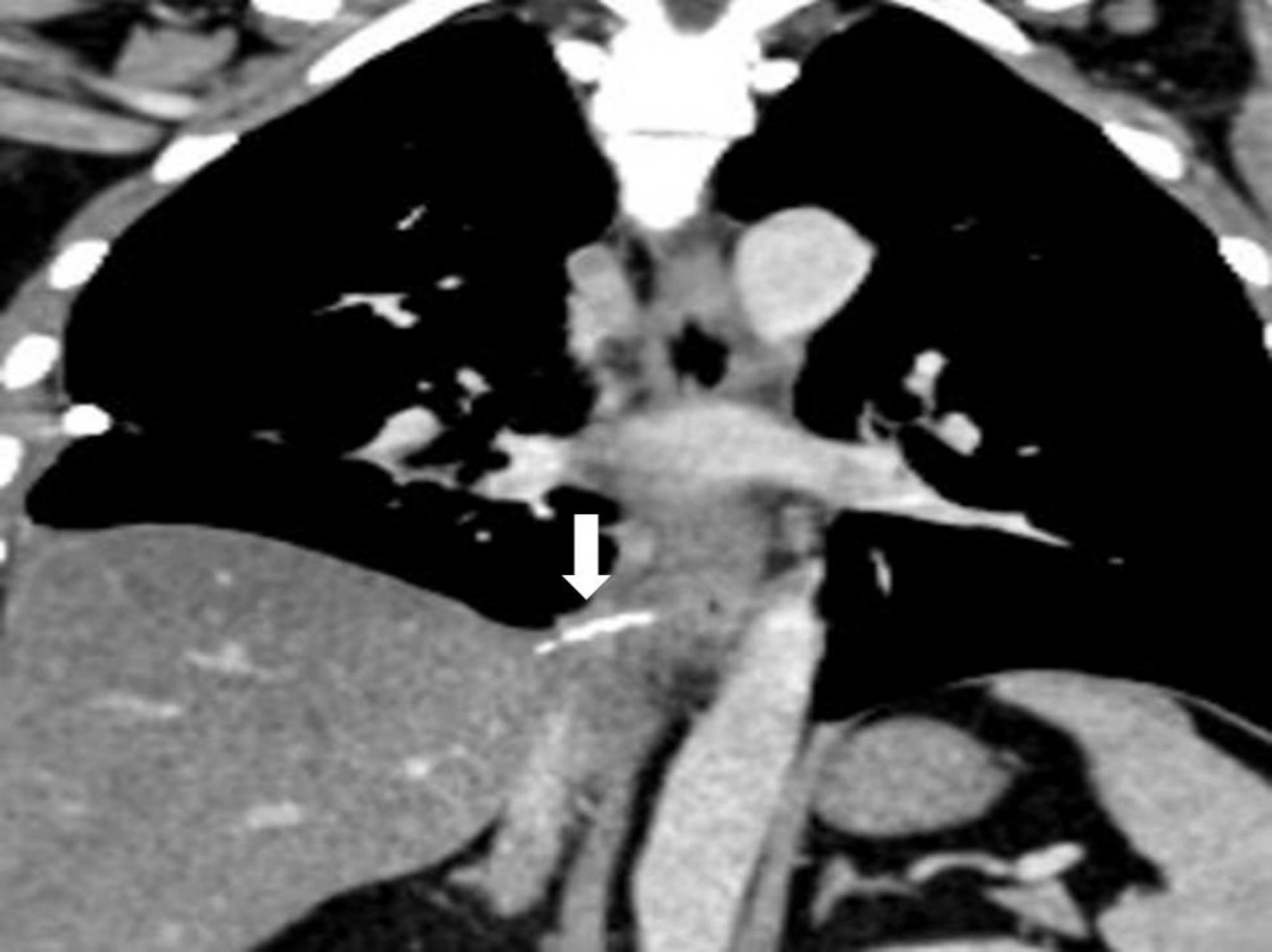
Coronal section showing the foreign body in the periesophageal region along the lower thoracic esophagus (white arrow)

A 38-year-old male presented to the surgery outpatient department with complaints of odynophagia and chest pain for two weeks. The symptoms had started after a meal when the patient felt the sensation of a foreign body in the throat, which he reportedly swallowed with a bolus of food. As the initial evaluation with neck and chest X-rays was reported as normal, he did not receive any specific intervention. However, in the third week after ingestion, he developed chest pain and fever. On evaluation with a contrast-enhanced thorax, chest, and abdomen scan, he was diagnosed with a right-sided pleural effusion and a suspected foreign body in the periesophageal region of the lower thoracic esophagus close to the inferior vena cava (Figures 1, 2).

From the hiatus region, the foreign body was located at approximately 5 cm cephalad. An intercostal drain was placed under image guidance and approximately 750 mL of serosanguinous pleural effusion was drained. An upper gastrointestinal contrast study did not show any contrast extravasation from the esophageal lumen. Upper gastrointestinal endoscopy also did not show any signs of recent perforation or a foreign body in the esophagus. An after adequate chest optimization, he underwent laparoscopic foreign body removal.

**Figure 3:**
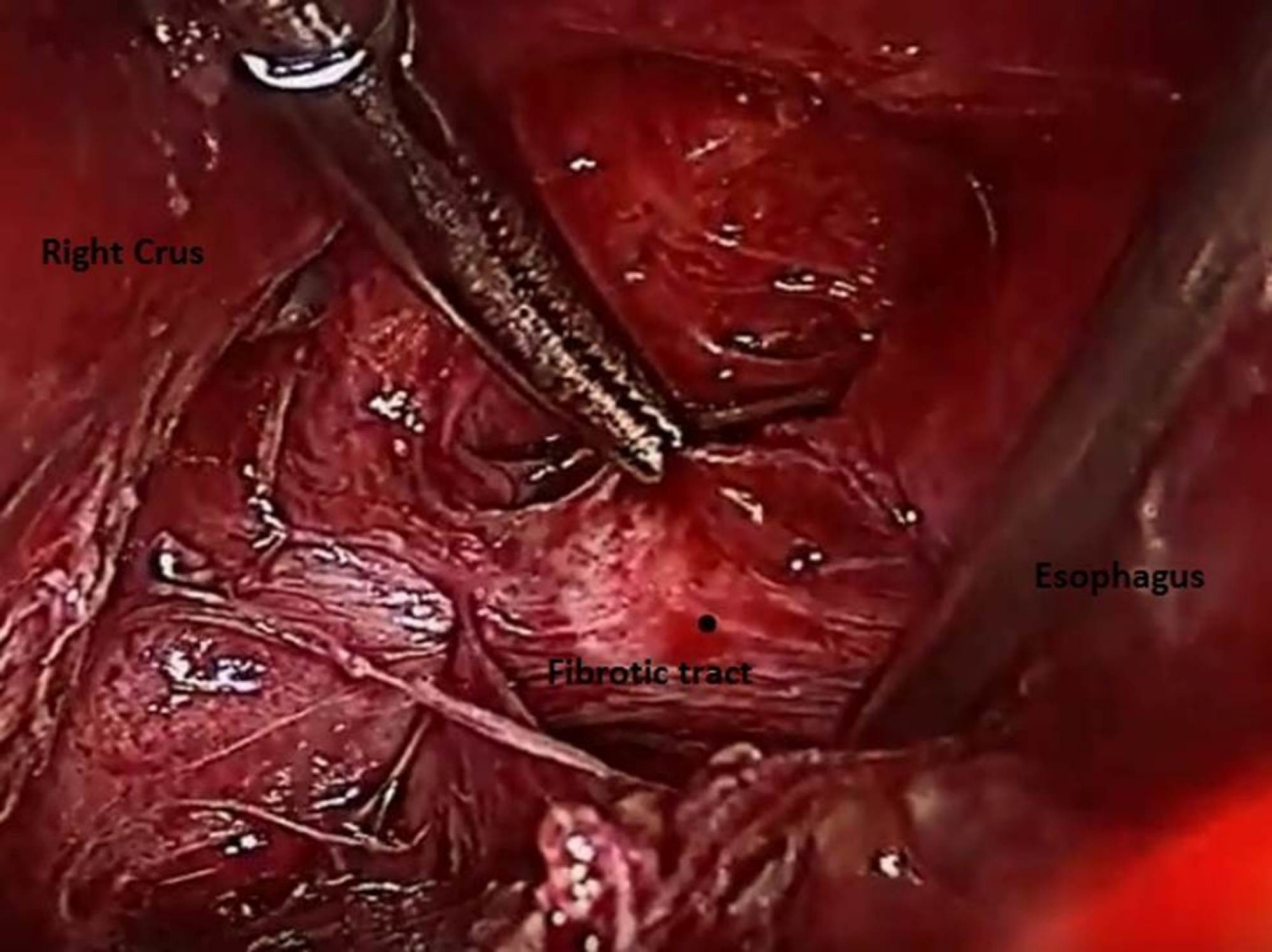
Dissection of the fibrotic tract which enclosed the foreign body

The procedure was done under general anesthesia in the supine split leg position. After initial access and the creation of a pneumoperitoneum through the left paramedian 12 mm port using an open method, five additional 5 mm ports were placed, including two midclavicular line ports on either side, a left subcostal port for assistance, and an epigastric port for liver retraction. The gastrohepatic ligament was divided to reach the right crus. The phrenoesophageal membrane was then opened to enter the mediastinum. The esophagus was then looped with an umbilical tape at the level of the hiatus and used for subsequent retraction. The right side of the esophagus was dissected meticulously, preserving the vagus nerves. At about 6 cm from the gastro esophageal junction, there was a fibrotic tract along the lateral aspect, which was then cut with scissors exposing the 2.7 cm long metallic foreign body (Figure 3).

**Figure 4:**
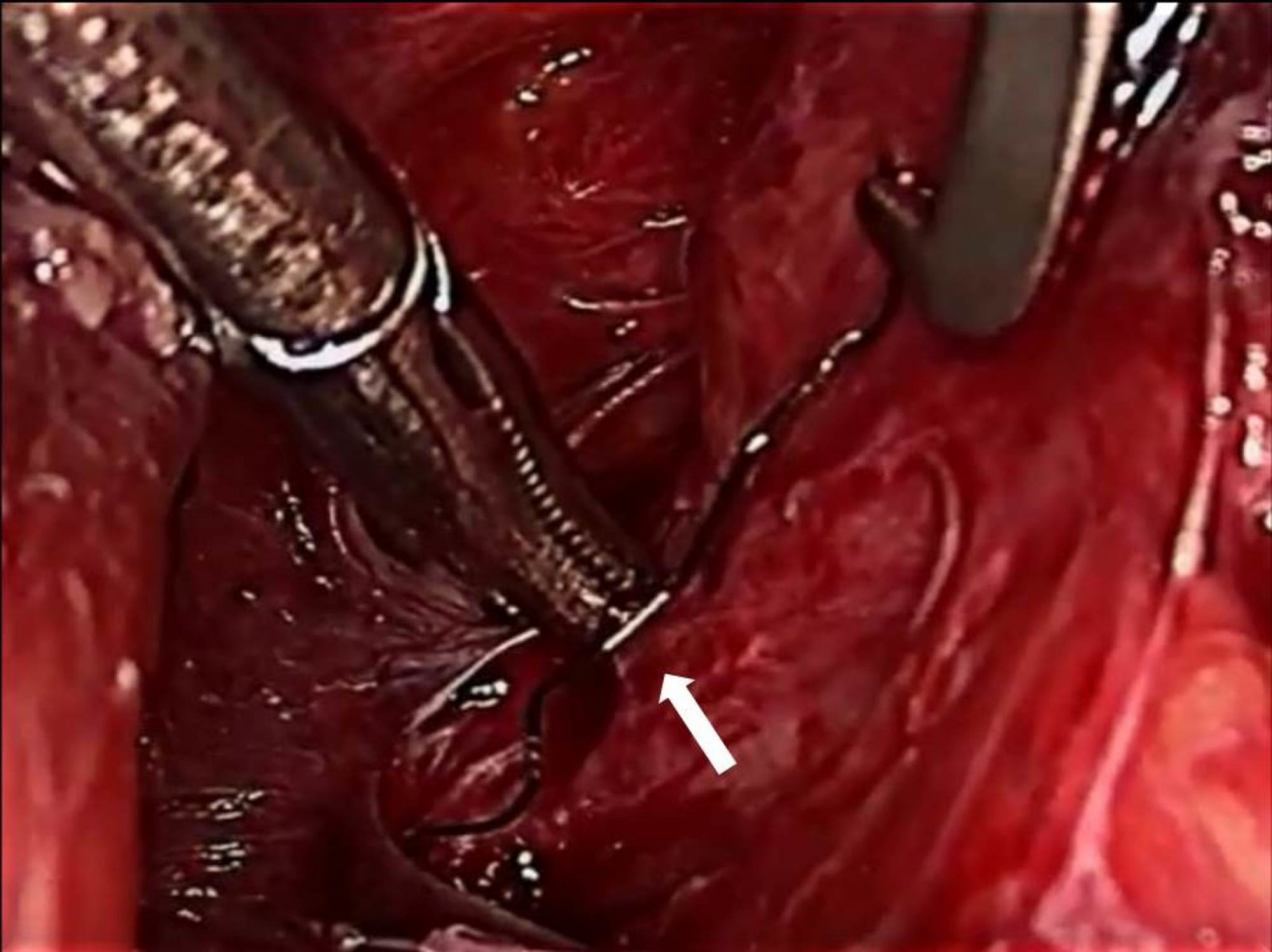
Foreign body being removed from the fibrotic tract (white arrow)

Right pleura were thickened and were not opened. The foreign body was then extracted and removed under vision (Figure 4).

The tract, which was seen extending to the esophagus, was clipped on the esophageal side using a hemolock. A thorough mediastinal lavage was given and a 16 Fr suction drain was placed in the paraesophageal region. An upper gastrointestinal oral contrast study that was done on the second postoperative day did not reveal any contrast leak, and the patient was started on an oral diet. He had an uneventful postoperative course and was discharged on the fifth postoperative day.

## Discussion

The surgical management of an esophageal foreign body becomes inevitable when there is a failure of the endoscopic approach or in the presence of mediastinal sepsis. When the esophagus is viable and the contamination is limited, primary esophageal repair with or without buttressing may be sufficient. Transesophageal migration of the foreign body into the mediastinum after a contained perforation in the esophagus is a rarer presentation. The foreign body in the mediastinum should be removed even in asymptomatic patients as there is a risk of adjacent organ and vascular injury. Traditionally, the transthoracic approach is used to remove an ingested foreign body with transesophageal migration into the mediastinum. Multiple reports have also described the thoracoscopic removal of the foreign body from the esophagus [7-9]. Thoracoscopy minimizes thoracotomy-related complications; however, it often requires single lung ventilation which is associated with pulmonary complications in the postoperative period [10].

The safety and feasibility of the laparoscopic transhiatal esophagectomy for lower thoracic esophageal tumors has been reported worldwide, including in the recent case series by Vageesh BG et al. [11]. The use of the laparoscopic approach for the removal of an esophageal foreign body had not been reported earlier. In the present patient, the laparoscopic approach was preferred over thoracoscopy, as the foreign body was located in the periesophageal region of the lower thoracic esophagus, close to the hiatus. Also, the patient did not have evidence of esophageal perforation or mediastinal sepsis at the time of presentation that precluded the need for transthoracic lavage and drainage. Thoracoscopy in patients with a history of intercostal drainage for pleural collection will be difficult due to pleural adhesions. A wide hiatal exposure, adequate esophageal traction, and meticulous periesophageal dissection can facilitate the removal of an esophageal foreign body with migration into the lower mediastinum. Also, the laparoscopic approach obviates the need for single lung ventilation and its associated morbidity. 

## Conclusions

The laparoscopic approach is feasible for the removal of an ingested foreign body with transesophageal migration into the lower mediastinum in the absence of significant mediastinal sepsis.
